# RASSF10 is an epigenetically inactivated tumor suppressor and independent prognostic factor in hepatocellular carcinoma

**DOI:** 10.18632/oncotarget.6654

**Published:** 2015-12-18

**Authors:** Fei Wang, Ying Feng, Peng Li, Kun Wang, Liang Feng, Yi-Fei Liu, Hua Huang, Yi-Bing Guo, Qin-Sheng Mao, Wan-Jiang Xue

**Affiliations:** ^1^ Department of General Surgery, Nantong University Affiliated Hospital, Nantong, Jiangsu, China; ^2^ Department of Pathology, Nantong University Affiliated Hospital, Nantong, Jiangsu, China; ^3^ Department of Surgical Comprehensive Laboratory, Nantong University Affiliated Hospital, Nantong, Jiangsu, China; ^4^ Department of General Surgery, Changzhou Wujin People's Hospital, Changzhou, Jiangsu, China

**Keywords:** RASSF10, hepatocellular carcinoma, DNA methylation, tumor suppressor, biomarker

## Abstract

Methylation of the Ras-association domain family 10 (RASSF10) promoter region correlates with clinicopathological characteristics and poor prognosis in several human cancers. Here, we examined RASSF10 expression in hepatocellular carcinoma (HCC) and its role in hepatocarcinogenesis. RASSF10 mRNA and protein levels were downregulated in both HCC cell lines and patient tissue samples. In patient tissues, low RASSF10 levels correlated with hepatocirrhosis, poor tumor differentiation, tumor thrombus and Barcelona Clinic Liver Cancer stage, and were indicative of increased tumor recurrence and reduced patient survival. Low RASSF10 expression was associated with promoter hypermethylation, which was in turn associated with polycyclic aromatic hydrocarbon and aflatoxin B1 exposure, but not DNA methyltransferase expression. Overexpression of RASSF10 in HCC cell lines suppressed cell growth and colony formation, and induced apoptosis by up- or down-regulating specific Bcl-2 family proteins. RASSF10 overexpression increased pro-apoptotic Bax and Bad levels, but decreased anti-apoptotic Bcl-2 and Bcl-xl expression. Overexpression also inhibited tumor formation in nude mice and reduced cell migration and invasion by inhibiting the epithelial-mesenchymal transition. RASSF10 knockdown promoted cell growth. Our results show that RASSF10 is frequently hypermethylated and down-regulated in HCC and can potentially serve as a useful biomarker predictive of HCC patient prognosis.

## INTRODUCTION

Hepatocellular carcinoma (HCC) is a major cause of mortality worldwide [1]. Of the approximately 782,500 new HCC cases annually, China accounts for nearly half [2, 3]. Genetic alterations and epigenetic modifications, especially DNA methylation, are both associated with HCC development and progression [4, 5]. HCC is likely to spread *via* hematogenous dissemination at an early stage [6]. Due to the absence of simple and effective diagnostic indicators and recognizable early symptoms, most HCC patients are diagnosed at an advanced stage, when surgery is no longer feasible, which results in a poor prognosis [7]. New strategies are therefore urgently needed for early HCC diagnosis, metastasis inhibition and treatment.

The Ras-association domain family 10 (RASSF10) gene is a candidate tumor suppressor gene (TSG) and the most recently discovered member of the RASSF family [8]. Located on chromosome 11p15.2, it has a CpG island of > 2 kb in its promoter region [9, 10]. Like other RASSF family members, hypermethylation of the RASSF10 promoter region, which inactivates the gene, is common across several cancers [11-24]. Moreover, methylation of the RASSF10 promoter region correlates with clinicopathological characteristics and a poor prognosis in several human cancers [21-24]. RASSF10 activates the P53 signaling pathway [23] and inhibits the Wnt/β-catenin signaling pathway [16], two major signaling cascades in HCC initiation and progression [25]. By modulating key signaling pathways, RASSF10 is essential for suppressing cell proliferation, regulating the cell cycle and inducing apoptosis [26]. RASSF10 is upregulated by JunD and PKA signaling upon contact inhibition [12], and its overexpression decreases cellular proliferation in glioma cell lines [24]. RASSF10 overexpression also potentiates docetaxel-induced tumor cell apoptosis, thereby increasing tumor cell sensitivity to chemotherapy [23]. However, our understanding of the function of RASSF10 in cancer is incomplete, and its role in hepatocarcinogenesis is unknown.

Here, we examined RASSF10 expression in HCC and its role in hepatocarcinogenesis. We found that hypermethylation of the RASSF10 promoter region downregulated its expression in HCC, and that RASSF10 expression is an independent prognostic factor for patient survival and tumor recurrence. RASSF10 hypermethylation was associated with polycyclic aromatic hydrocarbon (PAH) and aflatoxin B1 (AFB1) exposure in HCC tissues, and RASSF10 overexpression suppressed the growth of HCC *in vitro* and *in vivo*. These results indicate that RASSF10 is a potential therapeutic target and may be a useful biomarker of HCC prognosis.

## RESULTS

### RASSF10 is downregulated in human HCC tissue

**Figure 1:**
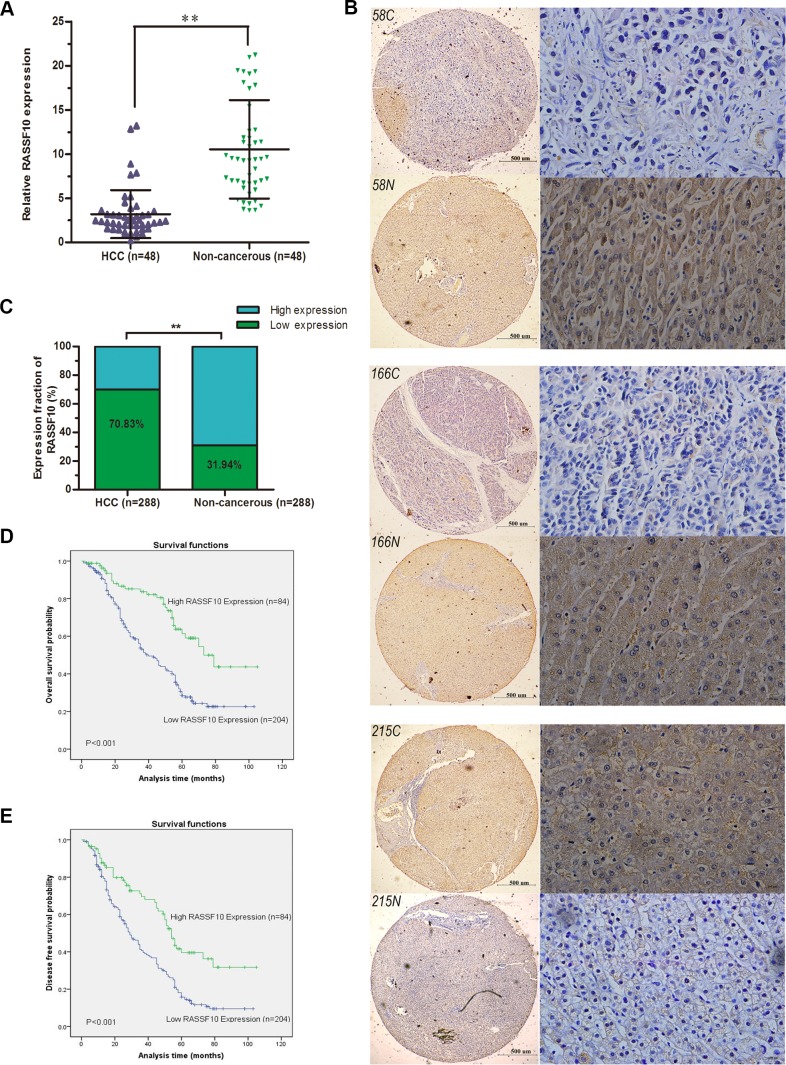
Downregulation of RASSF10 in HCC RASSF10 mRNA levels **A.** in the 48 paired HCC tissues were evaluated by qRT-RCR. Representative images **B.** of RASSF10 IHC staining in 288 paired HCC (58C, 166C and 215C) and matched non-cancerous tissue samples (58N, 166N and 215N) (left, Bar 500μm ×40, and right, Bar 100μm ×400). RASSF10 protein levels **C.** in 288-paired HCC and matched non-cancerous tissue were determined by IHC. Overall survival **D.** and disease free survival analysis **E.** of 288 HCC patients by Kaplan-Meier method. **P < 0.001.

To determine whether RASSF10 is downregulated in HCC, we first measured the expression of RASSF10 mRNA in fresh HCC tissue and matching non-cancerous liver samples by quantitative real-time polymerase chain reaction (qRT-PCR). In 66.7% (32/48) of tissue pairs, RASSF10 mRNA expression was lower in HCC tissue than in non-cancerous tissue (*P* < 0.001; Figure 1A). Next, we used tissue microarray (TMA) and immunohistochemistry (IHC) methods to examine RASSF10 protein expression in HCC and matching non-cancerous liver tissue. RASSF10 protein was localized mainly in the cytoplasm of HCC cells (Figure 1B). Low expression of RASSF10 was detected in 70.83% (204/288) of HCC tumors and 31.94% (92/288) of non-cancerous tissue samples (*P* < 0.001; Figure 1C). These results suggest that RASSF10 expression is downregulated in HCC.

### Low RASSF10 expression is associated with clinicopathological characteristics and reduced survival

To investigate the clinical significance of RASSF10 expression, we analyzed the relationship between RASSF10 protein expression status (low or high) and clinicopathological characteristics in HCC patients. We found that low RASSF10 expression was associated with tumor differentiation, hepatocirrhosis, Barcelona Clinic Liver Cancer (BCLC) stage and tumor thrombus. No correlation was found between low RASSF10 expression and age, gender, serum α-Fetoprotein (AFP), tumor size, liver function (Child-Pugh stage), tumor satellites or presence of an envelope (Table 1). A multivariate, unconditional logistic regression model also showed no correlation between RASSF10 expression and most pathological parameters. However, low RASSF10 expression was more likely in HCC patients with tumor thrombus (*P* = 0.001, odds ratio (OR) = 0.119, 95% confidence interval (CI): 0.036-0.397) and hepatocirrhosis (*P* < 0.001, OR = 0.321, 95% CI: 0.181-0.572).

**Table 1:** Relationship between RASSF10 expression and clinicopathological characteristics in HCC patients

Clinicopathological characteristics	*n*	Low expression	High expression	*χ*^2^	*P* value
**Total**	288				
**Gender**				0.934	0.361
Male	220	159(72.3%)	61(22.7%)		
Female	68	45(66.2%)	23(33.8%)		
**Age (years)**				1.413	0.246
≤52	146	108(74%)	38(26%)		
>52	142	96(67.6%)	46(32.4%)		
**Grade of differentiation**				10.566	0.005*
Low	117	72(61.5%)	45(38.5%)		
Middle	90	74(82.2%)	16(17.8%)		
High	81	58(71.6%)	23(28.4%)		
**Tumor diameter (cm)**				0.695	0.462
≤5	212	153(72.2%)	59(27.8%)		
>5	76	51(67.1%)	25(32.9%)		
**Liver function (Child-Pugh stage)**				1.339	0.316
A	235	168(69.4%)	72(30.6%)		
B or C	53	41(77.4%)	12(22.6%)		
**Hepatocirrhosis**				20.922	<0.001*
Absent	74	37(50.0%)	37(50.0%)		
Present	214	167(78.0%)	47(22.0%)		
**HBV infection**				2.635	0.111
Absent	110	84(76.4%)	26(23.6%)		
Present	178	120(67.4%)	58(32.6%)		
**Tumor thrombus**				19.654	<0.001*
Absent	231	150(64.9%)	81(35.1%)		
Present	57	54(94.7%)	3(5.3%)		
**AFP (ng/ml)**				0.218	0.697
≤20	131	91(69.5%)	40(30.5%)		
>20	157	113(72.0%)	44(28.0%)		
**BCLC stage**				5.172	0.031*
A	206	138(67%)	68(33.0%)		
B, C, or D	82	66(80.5%)	16(19.5%)		
**Envelope**				0.092	0.796
Absent	140	98(70.0%)	42(30.0%)		
Present	148	106(71.6%)	42(28.4%)		
**Tumor satellite**				0.281	0.589
Absent	245	175(71.4%)	70(28.6%)		
Present	43	29(67.4%)	14(32.6%)		

* *P* < 0.05.

Univariate analysis showed that HCC patients with low RASSF10 expression had shorter overall survival (OS) and disease-free survival (DFS) after curative resection than those with high RASSF10 expression (Figure 1D-1E). Moreover, multivariate analysis showed that RASSF10 protein expression was an independent predictor of OS and DFS (Table 2). Median OS and DFS were reduced in patients with low RASSF10 expression as compared to those with high expression (OS: 36.4 *vs*. 46.1 months, respectively, *P* = 0.002; DFS: 32.2 *vs*. 41.7 months, respectively, *P* = 0.003). Multivariate analysis also revealed that hepatocirrhosis and BCLC stage were associated with OS and that hepatocirrhosis and tumor thrombus were associated with DFS.

**Table 2:** Univariate and multivariate analysis of OS and DFS for HCC patients

Variable	OS			DFS		
	Univariate analysis	Multivariable analysis		Univariate analysis	Multivariable analysis	
	*P*>|z|	*P*>|z|	HR(95%CI)	*P*>|z|	*P*>|z|	HR(95%CI)
**RASSF10 expression**						
Low (*n* = 204) *vs*. high (*n* = 84)	0.008*	0.002*	0.510(0.335-0.778)	0.003*	0.003*	0.588(0.412-0.840)
**Gender**						
Male (*n* = 220) *vs*. female (*n* = 68)	0.270			0.643		
**Age (years)**						
≤52 (*n* = 146) *vs*. >52 (*n* = 142)	0.346			0.735		
**Grade of differentiation**						
Low (*n* = 117) *vs*. middle (*n* = 90) *vs*. high (*n* = 81)	0.985			0.614		
**Tumor diameter (cm)**						
≤5 (*n* = 212) *vs*. >5 (*n* = 76)	0.847			0.706		
**Liver function (Child-Pugh stage)**						
A (*n* = 235) *vs*. B or C (*n* = 53)	0.607			0.830		
**Hepatocirrhosis**						
Absent (*n* = 74) *vs*. present (*n* = 214)	0.018*	0.020*	1.728(1.091-2.737)	0.034*	0.019*	1.552(1.074-2.242)
**Hepatitis B virus**						
Absent (*n* = 110) *vs*. present (*n* = 178)	0.788			0.632		
**Tumor thrombus**						
Absent (*n* = 231) *vs*. present (*n* = 57)	0.025*	#		0.035*	0.008*	1.540(1.120-2.117)
**AFP (ng/ml)**						
≤20 (*n* = 131) *vs*. >20 (*n* = 157)	0.630			0.866		
**BCLC stage**						
I (*n* = 206) *vs*. II, III, or IV (*n* = 82)	0.009*	0.001*	1.741(1.262-2.401)	0.195		
**Envelope**						
Absent (*n* = 140) *vs*. present (*n* = 148)	0.752			0.773		
**Tumor satellite**						
Absent (*n* = 245) *vs*. present (*n* = 43)	0.615			0.681		

* *P* < 0.05

# Tumor thrombus is a very important parameter in the BCLC classification, and should be excluded in multivariate analyses.

### RASSF10 transcriptional expression is regulated by promoter hypermethylation

**Figure 2:**
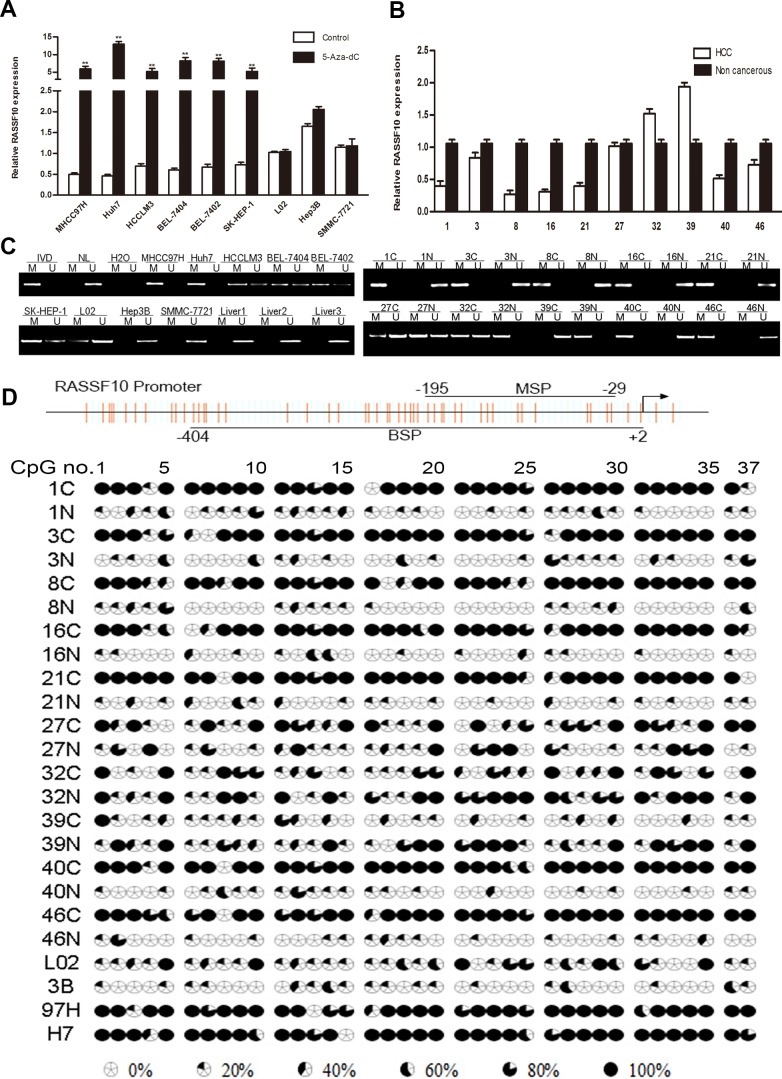
Downregulation of RASSF10 by promoter hypermethylation Analysis of RASSF10 expression levels **A.** in eight HCC cells with or without 5-aza-dC treatment was determined by qRT-PCR. RASSF10 expression **B.** in 10 matched pairs of non-cancerous liver (N) and HCC (C) tissues were analyzed by qRT-PCR. RASSF10 methylation status **C.** in 8 HCC cell lines and 3 cases of normal liver tissues, and in 10 matched pairs of non-cancerous liver (N) and HCC (C) tissues was analyzed by MSP. M and U: amplification using primers specific for methylated and unmethylated DNA, respectively. *In vitro* methylated DNA (IVD) and normal peripheral lymphocyte DNA (NL) were used as methylated and unmethylated controls. H_2_O was used as a blank control. BSPof RASSF10 promoter region (−404bp to +2bp) **D.** in 10 matched pairs of non-cancerous liver (N) and HCC (C) tissues, and in 3 HCC cell lines (3B: Hep3B, 97H: MHCC97H, H7: Huh7). The CpG island studied by MSP is indicated by a horizontal line marked with MSP, spanning 156 bp. The CpG island studied by BSP is indicated by a horizontal line marked with BSP. **P < 0.001.

To examine the expression of RASSF10 in HCC cells, we compared a normal human liver cell line (L02) with eight HCC cell lines (Huh7, HCCLM3, MHCC97H, BEL7404, SMMC-7721, SK-HEP-1, Hep3B, and BEL7402). The eight HCC cell lines expressed different levels of RASSF10 (Figure 2A). When compared with the L02 cell line, six HCC cell lines (MHCC97H, Huh7, HCCLM3, BEL7404, BEL7402, and SK-HEP-1) showed lower RASSF10 transcriptional expression and two (Hep3B and SMMC-7721) showed higher RASSF10 transcriptional expression. To determine whether promoter hypermethylation is involved in the expression of RASSF10, we treated all nine cell lines with the demethylating agent 5-aza-2′-deoxycytidine (5-aza-dC). The treatment increased RASSF10 expression in MHCC97H, Huh7, HCCLM3, BEL7404, BEL7402, and SK-HEP-1 cells (all *P* < 0.001; Figure 2A), suggesting that hypermethylation of the RASSF10 promoter impedes transcription. Using methylation-specific PCR (MSP) to examine the promoter region of RASSF10, we found complete methylation in Huh7 and MHCC97H cells and partial methylation in HCCLM3, BEL7404, BEL7402, and SK-HEP-1 cells (Figure 2C). Therefore, promoter methylation correlated with reduced RASSF10 expression. To further assess methylation density, we performed bisulfite sequencing PCR (BSP) of the CpG island of the RASSF10 promoter (nucleotides −404 to +2) spanning 37 CpG sites in MHCC97H, Huh7, and Hep3B cells. Five PCR clones of each specimen were sequenced (Figure 2D). We found that the MSP results correlated with the BSP results, as the samples in which the frequency of RASSF10 promoter methylation was < 20% were negative according to MSP (Figure 2D). Genomic sequencing was performed to exclude the possibility of RASSF10 mutation. No RASSF10 sequence variants were identified in MHCC97H and Huh7 cell lines or fresh HCC tissues. These results suggest that RASSF10 expression is mainly altered by promoter region methylation in HCC cell lines.

### RASSF10 expression is negatively correlated with RASSF10 promoter hypermethylation

To determine whether RASSF10 hypermethylation was related to its low expression, MSP was used to examine RASSF10 methylation in HCC and matching non-cancerous liver tissue samples, and normal liver tissue samples. Methylation was found in 64.58% (31/48) of HCC samples and 22.91% (11/48) of non-cancerous samples, but no methylation was found in normal liver samples (0/5). Of the 32 HCC samples with reduced RASSF10 expression as described above, methylation was found in 25 samples (Figure 2B-2C). Reduced RASSF10 transcriptional expression was negatively correlated with RASSF10 promoter hypermethylation (*r* = −0.400, *P* = 0.005). BSP was used to further examine methylation density; we found RASSF10 promoter methylation in 62.89% of HCC samples and 24.81% of non-cancerous samples (*P* < 0.001; Figure 2D).

### RASSF10 promoter hypermethylation is unrelated to DNA methyltransferase expression in HCC tissue

**Figure 3:**
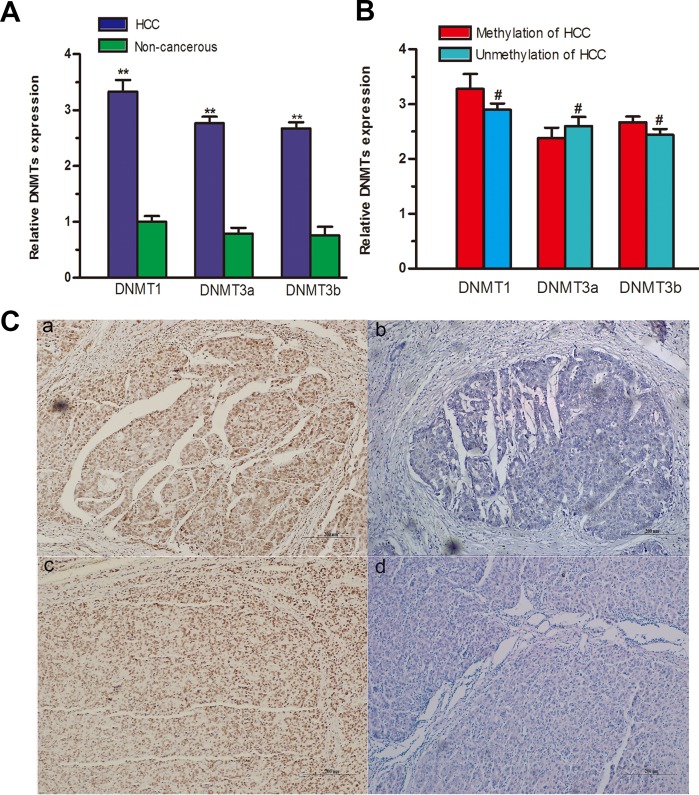
Relationships between RASSF10 promoter hypermethylation, DNMTs, and environmental carcinogen-DNA adducts DNMT mRNA levels **A.** were evaluated in 48 paired fresh HCC tissues by qRT-RCR. The expression of DNMT mRNAs **B.** was compared between methylated and unmethylated HCC groups. Immunoreactivity of AFB1-DNA and PAH-DNA adducts **C.** appeared as a dark brown nuclear stain in tumor cells, showed in a and c, respectively (Bar 200μm ×100), and no evidence of adducts is visible in b and d (Bar 200μm ×100). **P < 0.001, #*P* > 0.05.

To determine whether the expression of DNA methyltransferases (DNMTs) may contributes to RASSF10 promoter hypermethylation in HCC, we measured the expression of DNMT mRNA in fresh HCC tissue using qRT-PCR. Higher DNMT1 (*P* < 0.001), DNMT3a (*P* < 0.001), and DNMT3b (*P* < 0.001) mRNA levels were observed in HCC samples than in matching non-cancerous samples (Figure 3A). However, neither DNMT mRNA nor DNMT protein levels correlated with RASSF10 DNA hypermethylation status in HCC (*P* > 0.05; Figure 3B and Figure S1A-S1B).

### RASSF10 hypermethylation is associated with AFB1 and PAH exposure

To understand the relationship between RASSF10 gene methylation and environmental factors in HCC, we investigated potential hepatocarcinogenesis risk factors. Using immunostaining of PAH-DNA and AFB1-DNA adducts (Figure 3C), we found that RASSF10 methylation was more frequently associated with PAH-DNA-positive or AFB1-DNA-positive HCC tissue samples (Table 3). However, there was no correlation between RASSF10 methylation status and other factors such as gender, age, hepatitis virus B (HBV) status, smoking, or alcohol consumption. A multivariate, unconditional logistic regression model revealed more frequent RASSF10 methylation in patients with PAH-DNA-positive (*P* = 0.020, OR = 5.072, 95% CI: 1.297-19.839) or AFB1-DNA-positive (*P* = 0.043, OR = 4.125, 95% CI: 1.044-16.299) HCC tissue, but there was no association between RASSF10 hypermethylation and other potential risk factors.

**Table 3:** Correlations between RASSF10 methylation status and potential risk factors

Potential risk factors	*n*	RASSF10 methylation status		*χ*^2^	*P*-value
		Methylated(%)	Unmethylated(%)		
**Total**	48				
**Gender**				0.730	0.521
Male	33	20(60.6%)	13(39.4%)		
Female	15	11(73.3%)	4(26.7%)		
**Age (years)**				1.255	0.367
≤50	25	18(72.0%)	7(28.0%)		
>50	23	13(56.5%)	10(43.5%)		
**HBV**				1.560	0.228
−	17	9(52.9%)	8(47.1%)		
+	31	22(71.0%)	9(29.0%)		
**Cigarette smoking**				0.152	0.761
Non-smoker	18	11(61.1%)	7(38.9%)		
Smoker	30	20(66.7%)	10(33.3%)		
**Alcohol consumption**				1.964	0.221
Non-drinker	29	21(72.4%)	8(27.6%)		
Drinker	19	10(52.6%)	9(47.4%)		
**AFB1-DNA**				6.305	0.025*
-	17	7(41.2%)	10(58.8%)		
+	31	24(77.4%)	7(22.6%)		
**PAH-DNA**				7.704	0.007*
-	21	9(42.9%)	12(57.1%)		
+	27	22(81.5%)	5(18.5%)		

* *P* < 0.05.

### RASSF10 overexpression inhibits HCC cell growth *in vitro* and *in vivo*

**Figure 4:**
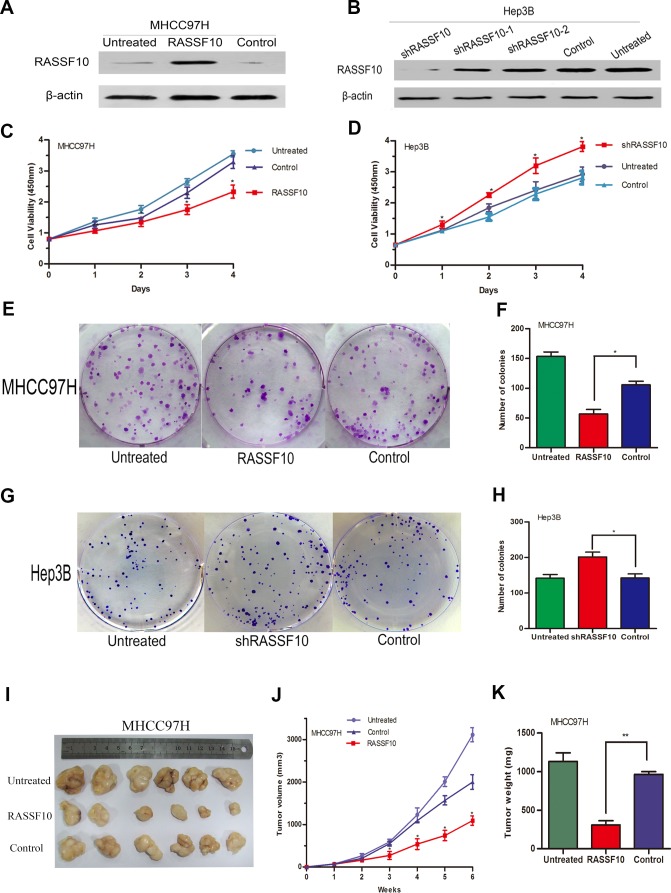
Function of RASSF10 as a tumor suppressor *in vitro* and *vivo* RASSF10 protein levels **A.** are shown in western blots of lentivirus-transfected MHCC97H cells. One subline expressed RASSF10 stably, and the control expressed empty lentiviral plasmid. MHCC97H cells with no lentiviral transfection served as the untreated control. RASSF10 protein levels **B.** are shown in western blots after plasmids containing three different short hairpin RNAs (shRASSF10, shRASSF10-1 and shRASSF10-2) were transiently transfected into Hep3B cells. The control expressed empty plasmid containing a random sequence of short hairpin RNA. Hep3B cells with no plasmid transfection served as the untreated controls. CCK-8 **C.**, **D.** and colony formation assays were employed to detect the effect of RASSF10 on the growth of the recombinant MHCC97H **E.**, **F.** and Hep3B **G.**,**H.** cell lines, Data are plotted as the mean ± SD of three independent experiments. Size, volume, and weight of tumors from mice treated with the recombinant MHCC97H cells **I.**, **J.**, and **K.** Points and Bars: mean of 6 mice. *P < 0.05, **P < 0.001.

To investigate the biological significance of RASSF10 expression in the development and progression of HCC, we performed gain- and loss-of-function experiments in HCC cells. We stably overexpressed RASSF10 in low-expressing MHCC97H and Huh7 cell lines using lentiviruses, and randomly selected two sublines (RASSF10 and RASSF10-2) to study. By transfecting a plasmid containing three different short hairpin RNAs (shRNAs), we transiently abrogated RASSF10 expression in the high-expressing Hep3B cell line. Western blots showed altered RASSF10 expression in these cell lines (Figure 4A-4B, Figure 7A and Figure S2A). The effect of RASSF10 alteration on HCC cell growth was assessed using a CCK8 assay. We found that transfected MHCC97H and Huh7 cells exhibited lower proliferation rates than control cells (Figure 4C, Figure 7B and Figure S2B), but that transfected Hep3B cells exhibited a higher proliferation rate (Figure 4D). In a colony formation assay, we observed fewer colonies with transfected MHCC97H and Huh7cells than with control cells (all *P* < 0.05; Figure 4E-4F, Figure 7C-7D and Figure S2C), but more colonies with transfected Hep3B cells (*P* = 0.005; Figure 4G-4H).

To assess the function of RASSF10 *in vivo*, we overexpressed RASSF10 in MHCC97H cells, implanted these cells into nude mice, and monitored tumor growth. After six weeks, volumes (Figure 4I) and weights (*P* < 0.001; Figure 4K) of RASSF10-overexpressing tumors were decreased as compared to controls. Furthermore, growth in RASSF10-overexpressing tumors was slower than that in controls (Figure 4J).

### RASSF10 induces apoptosis of HCC cells *via* Bcl-2 family proteins

Fluorescence-activated cell sorting (FACS) was performed to evaluate the effects of RASSF10 expression on cell cycle progression. Overexpression of RASS10 in MHCC97H and Huh7 cells or knockdown of RASS10 in Hep3B cells had no effect on DNA content during G1/G0-, S-, or G2/M-phases, suggesting that RASSF10 level does not affect HCC cell cycle (data not shown).

**Figure 5:**
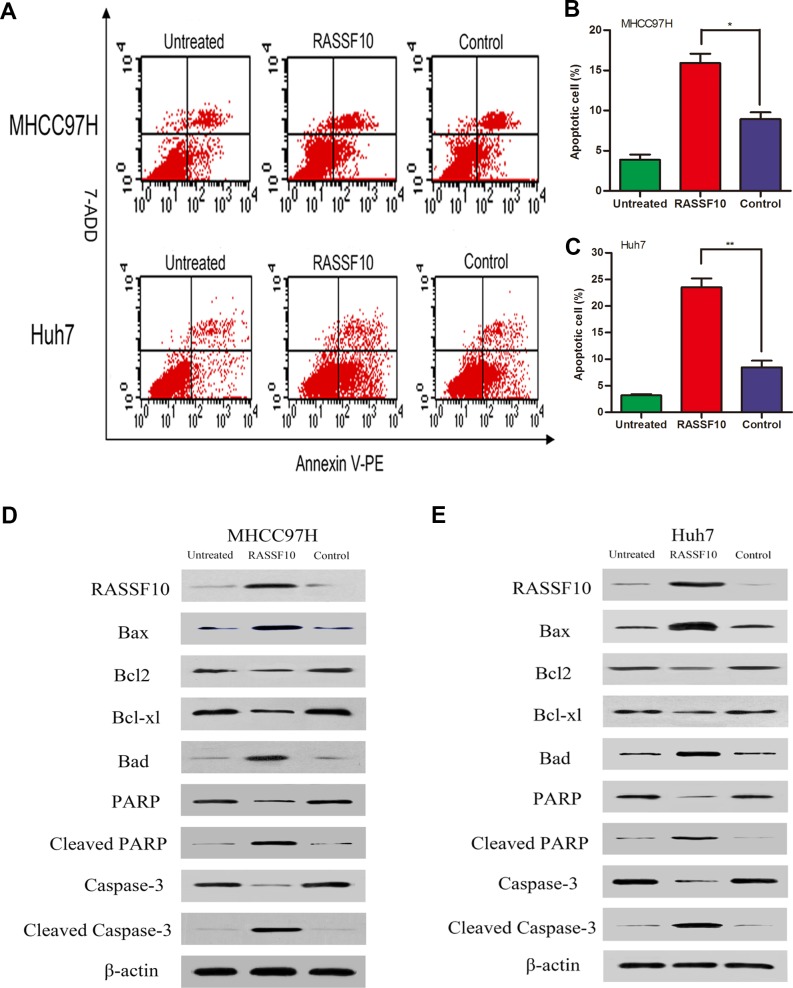
Effect of RASSF10 on apoptosis of HCC cells The early stage apoptosis cells were detected for Annexin V-PE+/7-AAD- **A.** One subline expressed RASSF10 stably, and the control expressed empty lentiviral plasmid. MHCC97H and Huh7 cells with no lentiviral transfection served as untreated controls. Percentage of apoptosis cells in recombinant MHCC97H **B.** and Huh7 **C.** cells. Quantitative analyses of apoptotic cell numbers are plotted as the mean ± SD of 3 independent experiments. Western blot analysis of RASSF10, Bax, Bad, Bcl-2, Bcl-xl, PARP, cleaved PARP, caspase-3 and cleaved caspase-3 expression in recombinant MHCC97H **D.** and Huh7 **E.** cells. **P < 0.001.

Next, we investigated the effect of RASSF10 overexpression on cell apoptosis using flow cytometry and Annexin V/7-AAD. We found that the early apoptosis rates (Annexin V-PE+/7-AAD−) of RASSF10-transfected MHCC97H and Huh7 cells were higher than those of control cells (all *P* < 0.05; Figure 5A-5C and Figure S2F). As the apoptosis rate of untreated Hep3B cells was only 3.04%, we did not measure changes in apoptosis rate after knockdown of RASSF10 in this line.

We also analyzed whether RASSF10-induced HCC cell apoptosis was related to changes in the expression of Bcl-2 family proteins, PARP, and caspase-3 in MHCC97H and Huh7 cells. Western blotting indicated that RASSF10 overexpression increased pro-apoptotic Bax and Bad expression but decreased anti-apoptotic Bcl-2 and Bcl-xl expression (Figure 5D-5E). Cleaved PARP and cleaved caspase-3 expression were also increased. These results suggest that RASSF10 may induce apoptosis in HCC cells by affecting the expression of Bcl-2 family proteins.

### RASSF10 controls HCC cell migration and invasion by inhibiting EMT

**Figure 6:**
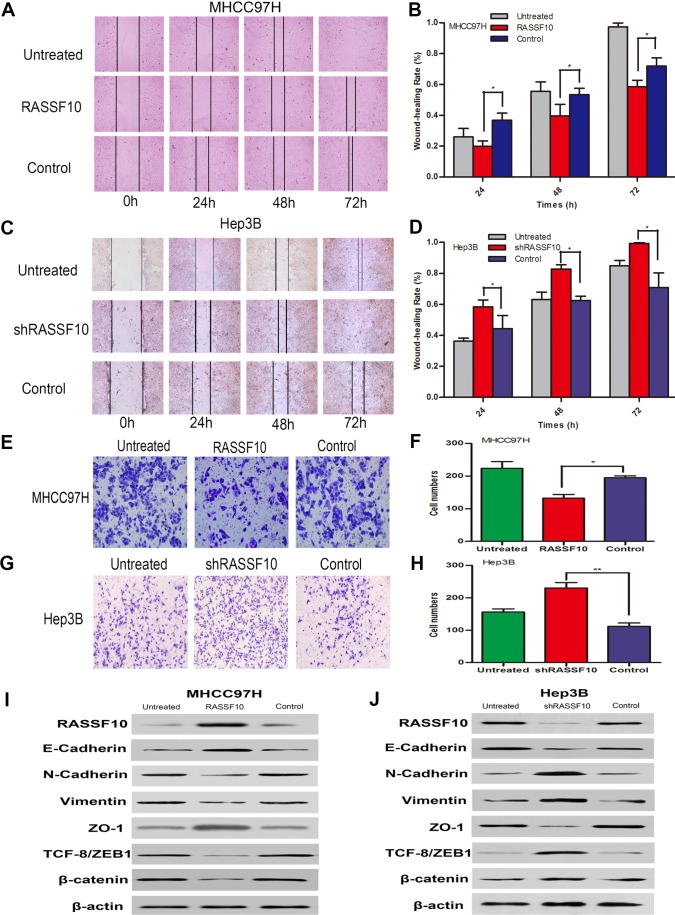
Influence of RASSF10 on HCC cell migration and invasion and EMT Wound healing assays **A.** and **B.** and invasion behavior **E.** and **F.** in lentiviral transfected MHCC97H cells. One subline expressed RASSF10 stably, and the control expressed empty lentiviral plasmid. MHCC97H cells with no lentiviral transfection served as untreated controls. Data are plotted as the mean ± SD of three independent experiments. Wound healing assays **C.** and **D.** and invasion behavior **G.** and **H.** after plasmids containing one shRNA (shRASSF10) was transiently transfected into Hep3B cells. The control expressed empty plasmid containing a random sequence of shRNA. Hep3B cells with no plasmid transfection served as untreated controls. Data are plotted as the mean ± SD of three independent experiments. Western blot analysis of RASSF10, E-cadherin, N-cadherin, Vimentin, ZO-1, ZEB and β-catenin expression in the recombinant MHCC97H **I.** and Hep3B **J.** cells. *P < 0.05, **P < 0.001.

**Figure 7:**
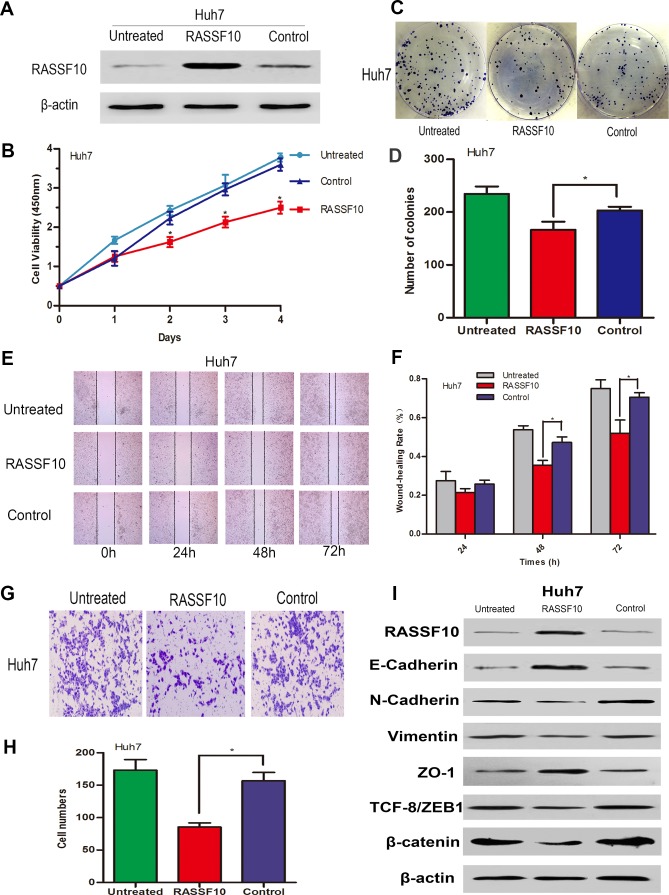
Influence of RASSF10 on Huh7 cell growth, migration, invasion and EMT RASSF10 protein levels **A.** in lentivirus-transfected Huh7 cells. One subline expressed RASSF10 stably, and the control expressed empty lentiviral plasmid. Huh7 cells with no lentiviral transfection served as untreated controls. Proliferation **B.** and colony formation **C.** and **D.** of the recombinant Huh7 cells. Wound healing **E.** and **F.** and invasion behavior **G.** and **H.** of the recombinant Huh7 cells. Western blot analysis of RASSF10, E-cadherin, N-cadherin, Vimentin, ZO-1, ZEB and β-catenin expression after the recombinant Huh7 cells **I.** All experiments were repeated at least three times. Data are plotted as the mean ± SD. *P < 0.05.

Finally, we examined the effect of RASSF10 expression on HCC cell migration and invasion. Wound healing assays showed that RASSF10 overexpression in MHCC97H and Huh7 cells was associated with slower healing rates (Figure 6A-6B, Figure 7E-7F and Figure S2D). Similarly, Matrigel transwell assays demonstrated that transfected MHCC97H and Huh7 cells exhibited reduced invasion compared with control cells (Figure 6E-6F, Figure 7G-7H and Figure S2E). Consistent with these findings, RASSF10 knockdown in Hep3B cells was associated with faster rates of wound healing and increased invasion compared with control cells (Figure 6C-6D, and Figure 6G-6H).

To investigate whether RASSF10 expression suppresses HCC cell migration and invasion by inhibiting the epithelial-mesenchymal transition (EMT), we examined the expression of EMT biomarkers in transfected HCC cell lines *via* western blotting. RASSF10 overexpression increased E-cadherin and ZO-1 protein levels, but decreased N-cadherin, β-catenin, Vimentin, and TCF-8/ZEB1 in MHCC97H and Huh7 cells (Figure 6I and Figure 7I). RASS10 knockdown decreased E-cadherin and ZO-1 protein levels, but increased N-cadherin, β-catenin, Vimentin, and TCF-8/ZEB1 in Hep3B cells (Figure 6J). Taken together, these results suggest that RASSF10 controls HCC cell migration and invasion by inhibiting EMT.

## DISCUSSION

The purpose of this study was to reveal the function of RASSF10 in hepatocarcinogenesis. We observed low levels of RASSF10 mRNA and protein in most HCC primary surgical samples and cell lines, suggesting that HCC tumorigenesis is associated with reduced RASSF10 expression. The low expression correlated with both hepatocirrhosis and tumor thrombus. Furthermore, following curative resection, both OS and DFS were lower in HCC patients with low RASSF10 expression, indicating that low RASSF10 expression may serve as a molecular biomarker of poor prognosis.

In the present study, RASSF10 was frequently hypermethylated in HCC tissue, resulting in its low expression, as has been reported for other cancers [10-23]. Although the mechanisms underlying DNA hypermethylation in HCC remains uncertain [27], three known catalytically active DNMTs have been identified: DNMT1, DNMT3a and DNMT3b. DNMT expression correlates with DNA hypermethylation status in other tumors, including acute myelocytic leukemia and bladder carcinoma [28, 29]. However, previous HCC studies report no relationship between DNMT expression and altered DNA methylation [30-33]. Most of these earlier studies used small samples, and levels of DNMT expression were examined using semi-quantitative RT-PCR.

Here, we used 48 paired HCC and non-cancerous liver tissue samples with qRT-PCR. We found that HCC samples had increased levels mRNAs encoding the three DNMTs, though these levels were unrelated to the patterns of RASSF10 methylation. We also found, however, that RASSF10 expression was increased after treating HCC cell lines with 5-aza-dC, a DNMT1 inhibitor. These results indicate that upregulated DNMT mRNA expression may not be sufficient to suppress RASSF10 expression in HCC, and additional strategies, such as viral infection to alter demethylase activity, may be necessary to specify the targeted cytosine residue for methylation [30, 33, 34]. Increased DNMT mRNA expression may also serve a function in hepatocarcinogenesis other than merely altering DNA methylation [35]. Another possible explanation is that in contrast to the limited types of cells that exist in cell lines, diverse cell types present in HCC tissues *in vivo* may affect the overall level of methylase expression. In addition, different splice variants of DNMT mRNA may encode different DNMT protein isoforms [36]. Lastly, DNMTs may suppress target gene expression by recruiting other histone modification proteins and affecting chromatin structure without changing the methylation status of target genes [37].

Environmental factors can also influence DNA methylation status by preventing or promoting methylation, thereby modifying HCC risk [38, 39]. However, the link between RASSF10 gene hypermethylation and environmental factors is currently unknown. We found that RASSF10 gene methylation correlated with AFB1-DNA and PAH-DNA adduct levels in HCC tissue but was unrelated to patients’ HBV status, smoking or drinking. Earlier studies reported that hypermethylation of TSG promoter regions, including those of RASSF1A and p16*^INK4A^*, correlate with AFB1-DNA and PAH-DNA adduct levels [40, 41], which suggests exposure to environmental carcinogens may alter the methylation status of key genes involved in hepatocarcinogenesis. As carcinogens, AFB1 and PAHs, especially benzo(a)pyrene, invade and erode target cells and then form carcinogen-DNA adducts. These adducts cause DNA damage and chromosomal aberrations that alter the expression of oncogenes and TSGs. In addition, adducts can induce hepatic oxidative damage, which may also influence methylation [42]. HBV infection is closely related to TSG and HBx-DNMTs-p16*^INK4A^* promoter hypermethylation, suggesting a possible mechanism for tumorigenesis in HCC [43]. However, our results indicate no relationship between RASSF10 methylation and HBV infection in HCC. This discrepancy between studies may be due to genetic or environmental differences, or the fact that we did not examine HBx proteins in the present study. Thus, additional studies with larger sample sizes will be required to verify our findings.

Because RASSF10 was shown to activate P53 signaling in colorectal cancer [23], we examined the expression of Bcl-2 family proteins in HCC. We found increased pro-apoptotic Bax and Bad expression but decreased anti-apoptotic Bcl-2 and Bcl-xl expression, suggesting RASSF10 overexpression suppresses HCC cell growth by up- or downregulating Bcl-2 family proteins. As RASSF10 may serve a tumor suppressor function, reversal of RASSF10 suppression could be a promising approach for HCC therapy.

We found that RASSF10 knockdown in HCC cells led to enhanced migration and invasion, and low RASSF10 expression in HCC was associated with tumor thrombus. Therefore, RASSF10 may act to suppress HCC cell migration and invasion. We found that RASSF10 upregulated E-cadherin and ZO-1 and downregulated mesenchymal markers such as Vimentin and N-Cadherin, contributing to suppression of EMT and attenuation of HCC cell migration and invasion. We also found that RASSF10 overexpression reduced β-catenin expression (Figure 6I, Figure 7I and Figure S3A-S3B). Wei and other investigators demonstrated that RASSF10 overexpression inhibits gastric carcinoma cell growth by suppressing Wnt/β-catenin signaling [16]. Furthermore, recent studies show that Wnt/β-catenin signaling is closely related to EMT [44]. We therefore speculate that RASSF10 overexpression inhibits EMT by inhibiting the Wnt/β-catenin pathway, thereby reducing HCC cell migration and invasion.

In conclusion, we found that RASSF10 is downregulated in HCC, leading to more aggressive tumor behavior and predicting poor patient outcome after curative resection. RASSF10 expression appears to be regulated by promoter region hypermethylation, which is correlated with PAH and AFB1 contamination. RASSF10 may suppress HCC cell growth by promoting cell apoptosis and by reducing HCC cell migration and invasion by inhibiting EMT. This suggests RASSF10 could serve as a useful biomarker predictive of patient prognosis and act as a target for HCC treatment.

## MATERIALS AND METHODS

### Cell lines and cell culture

The HCCLM3 and MHCC97H cell lines were gifts from the Liver Cancer Institute, Zhong Shan Hospital (Shanghai, China). One normal hepatocyte cell line (L02) and six HCC cell lines (Huh7, BEL7404, SMMC7721, SK-HEP-1, Hep3B, and BEL7402) were purchased from GeneChem (Shanghai, China) and cultured in medium recommended by GeneChem.

### Patients and tissue samples

HCC tissue and matching non-cancerous liver tissue samples were collected from HCC patients (*n* = 48) undergoing curative resection between June 2013 and June 2014 at the Affiliated Hospital of Nantong University (Jiangsu, China). Samples were used for qRT-PCR, MSP, BSP and IHC. Tissue samples were also collected from HCC patients (*n* = 288) undergoing curative resection between January 2004 and December 2009 at the same hospital and were used for TMA with a Tissue Microarray System (Quick-Ray, UT06, UNITMA, Korea). For the latter patients, follow-up was completed by December 2014, and the median follow-up period was 58 months (range: 3-105 months). These patients were monitored using abdominal ultrasonography, AFP tests, chest X-ray, magnetic resonance imaging, and/or computed tomography scans every 3-6 months after surgery. Overall survival and disease-free survival were calculated as previously described [45]. Two pathologists independently performed histopathological examination of samples using the Edmondson grading system: G1, high differentiation; G2 and G3, middle differentiation; G4, low differentiation. All patients with clear HCC pathology had not received neoadjuvant chemotherapy, radiation therapy, or immunotherapy before surgery. Patient clinical information, including gender, age, tumor diameter, tumor differentiation, AFP level, liver cirrhosis, HBV infection, portal vein tumor thrombus, and BCLC stage, were obtained from medical records during the inpatient and follow-up periods. Additionally, normal liver tissue samples (*n* = 5) were obtained from patients with liver hemangioma at the Affiliated Hospital of Nantong University. Preoperative blood tests confirmed that these patients did not have viral infections, and postoperative pathology confirmed that they did not suffer from HCC or liver cirrhosis. The human research ethics committee of the Affiliated Hospital of Nantong University approved procedures, and written informed consent was obtained from the patients.

### RNA extraction and qRT-PCR

The use of RASSF10 primers, total RNA extraction, and qRT-PCR were carried out as previously described [19, 41]. Other primers were as follows: DNMT1, 5′-TTGGTGGATGAATGTGAGGA-3′ (forward) and 5′-TGGTGGCTGAGTAGTAGAGGAC-3′ (reverse); DNMT3a, 5′-GGCGTTAGTGACAAGAGGG-3′ (forward) and 5′-TGGACTGGGAAACCAAATA-3′ (reverse); DNMT3b, 5′-CAAACACTTCCCCATAAAGG-3′ (forward) and 5′-GCGTGAGTAATTCAGCAGGT-3′ (reverse); glyceraldehyde-3-phosphate-dehydrogenase, 5′-AGAAGGCTGGGGCTCATTTG-3′ (forward) and 5′-AGGGGCCATCCACAGTCTTC-3′ (reverse). Experiments were repeated in triplicate.

### TMA and IHC

TMA and IHC were performed as previously described [41, 46]. Deparaffinized sections (4 μm) were stained using an Autostainer Universal Staining System (LabVision, Kalamazoo, MI). For the IHC studies, the primary antibodies were as follows: anti-RASSF10 (sc-243928), anti-AFB1-DNA (sc-66016), anti-benzopyrene-7,8-diol-9,10-epoxide-DNA (sc-52625), anti-DNMT1 (sc-271729), anti-DNMT3a (sc-20703), and anti-DNMT3b (sc-130740) (Santa Cruz Biotechnology, Dallas, TX). Phosphate-buffered saline was used instead of primary antibody as a negative control. Immunostaining evaluations were performed independently by experimenters blinded to sample identity. The staining intensity was scored as follows: 0 (negative), 1 (weakly positive), 2 (moderately positive), and 3 (strongly positive). The percent positivity was also scored according to four categories 0 ( < 5%), 1 (5%-25%), 2 ( > 25%-50%), 3( > 50%-75%) and 4 ( > 75%). In cases where differences between duplicate tissue cores were observed, the higher score was considered to be the final score. The final expression scores of these proteins were calculated with the value of percent positivity score multiplied by staining intensity score, which ranged from 0 to 12. The degree of protein staining was quantified using a two-level grading system, and staining scores were defined as follows: low expression or negative (score≤2) and high expression or positive (score≥3).

### MSP and BSP

Genomic DNA extraction, MSP, and BSP were performed as previously described [16, 41]. The sense and antisense primers for the methylated sequences were FM-RASSF10 (5′-GGGTTTTGCGAGAGCGCG-3′) and RM-RASSF10 (5′-GCTAACAAACGCGAACCG-3′), respectively. The sense and antisense primers for the unmethylated sequences were FU-RASSF10 (5′-GGTTTTGTGAGAGTGTGTTTAG-3′) and RU-RASSF10 (5′-CACTAACAAACACAAACCAAAC-3′), respectively. Primer sequences of BSP were 5′-GATTTAGGATGTTTGTAATGYG-3′ (forward) and 5′-TCTCTTCCTAACAAATCCACAC-3′ (reverse). Thirty-seven CpG sites spanning the regions were evaluated.

### RASSF10 exon mutation detection

Extraction of genomic DNA from HCC cells and tissue was performed as described for MSP and BSP analyses. Primer sequences for PCR were as follows: 5′-GATTTAGGATGTTTGTAATGYG-3′ (forward) and 5′-TCTCTTCCTAACAAATCCACAC-3′ (reverse). Bidirectional cycle sequencing of PCR products was performed.

### 5-aza-dC treatment

HCC cell lines were treated with 10 μM 5-aza-dC (Sigma, Taufkirchen, Germany) for five days. Untreated cells were used as a negative control. After treatment, cell lines were harvested for qRT-PCR.

### Lentivirus and plasmid transfection

RASSF10 ectopic expression and negative control lentiviruses were purchased from GeneChem. Concentrated lentivirus was transfected into HCC cells (MHCC97H and Huh7) with a multiplicity of infection of 10 in the presence of polybrene (10 μg/ml). Overexpressing cells were selected and used for experiments. Three GV248 lentiviral particles containing shRNA sequences targeting the human RASSF10 gene and negative control plasmids containing a random sequence of shRNA (shNC) were also purchased from GeneChem. The three pooled shRNA sequences achieving the greatest knockdown were: shRASSF10(5′-CGTCAAGTCGGACTTGGATTACTCGAGTAATCCAAGTCCGACTTGACG-3′), shRASSF10-1(5′-CAGTACCAGCCTTTACATTGCTCGAGCAATGTAAAGGCTGGTACTGA-3′), and shRASSF10-2(5′-ACGTGCAGGACACTTACTTGGCTCGAGCCAAGTAAGTGTCCTGCACGT-3′). Hep3B cells were transfected with three different shRNA or shNC plasmids using Lipofectamine 2000 (Invitrogen, Carlsbad, CA) according to the manufacturer's instructions. Transfected cells were incubated for 48 h and then harvested. The efficiency of RNA interference was determined by western blot.

### Western blot analysis

Western blotting was performed as previously described [41]. Antibodies were diluted according to the manufacturer's instruction. Primary antibodies were as follows: anti-E-Cadherin (#3195), anti-N-Cadherin (#13116), anti-Vimentin (#5741), anti-TCF8/ZEB1 (#3396), anti-ZO-1 (#8193), anti-β-Catenin (#8480), anti-Bax (#5023), anti-Bad (#9239), anti-Bcl-xL (#2764), anti-Bcl-2 (#2870), anti-PARP (#9532), anti-Caspase-3(#9662), anti-β-Actin (#4970). (Cell Signaling Technology, Boston, MA).

### Cell proliferation and colony formation assays

Cell proliferation was assessed using the Cell Counting Kit-8 (CCK-8, Beyotime Institute of Biotechnology) according to the manufacturer's instructions. For the colony formation assay, cells were incubated for 2 weeks, stained with crystal violet, (Beyotime Institute of Biotechnology) and imaged using a camera (Nikon, Tokyo, Japan). Only positive colonies (diameter > 40 μm) were counted.

### *In vivo* tumorigenicity assay

Tumor cells (5×10^6^) were injected subcutaneously into the flanks of 4-week-old male Balb/c athymic nude mice (*n* = 6 per group). Mice were monitored once per week and euthanized six weeks later. Tumors were dissected and weighed, and tumor volume was calculated as V (volume, mm^3^) = 0.5×L (length, mm)×W^2^ (width, mm^2^). Animal experiments were performed using protocols approved by the Animal Center of the Medical College of Nantong University.

### Flow cytometry analysis

Cell cycle progression was examined as previously described [47]. For apoptosis analysis, HCC cells were collected and stained with annexin V-PE and 7AAD (BD Biosciences, San Diego, CA) as previously described [23]. The percentage of apoptotic cells was calculated using a flow cytometer (Beckman Coulter, Indianapolis, IN).

### Wound healing assay

Cells were plated in a 6-well plate and scratched with a 200-μl pipette tip after achieving nearly 90% confluence. The speed of wound closure was imaged with an inverted microscope (TE-2000S, Nikon) at 0, 24, 48, and 72 h.

### Matrigel invasion assay

A 24-well transwell plate with inserted chambers (8-μm pore size; Costar, New York, NY) was used to measure the invasiveness of cells. Inserts were coated with 60 μl Matrigel (BD Biosciences, East Rutherford, NJ). After incubation for 24 h, invading cells were fixed and stained with 0.1% crystal violet containing formaldehyde. Invading cells were imaged and counted in five randomly selected fields under an inverted microscope (TE-2000S, Nikon).

### Statistical analysis

Quantitative data are shown as mean ± standard error of the mean of at least three independent experiments. Statistical comparisons were performed with *χ^2^* tests using SPSS software (version 17.0; Chicago, IL). Factors contributing to low RASSF10 expression and RASSF10 methylation were identified using a multivariable, unconditional logistic regression model. Survival curves were calculated using the Kaplan-Meier method. Univariate and multivariate analyses were subsequently performed using a Cox regression model to identify independent risk factors. For all tests, a *P*-value of < 0.05 was considered statistically significant.

## SUPPLEMENTARY MATERIAL FIGURES



## References

[1] Shiraha H, Yamamoto K, Namba M (2013). Human hepatocyte carcinogenesis (review). International journal of oncology.

[2] Torre LA, Bray F, Siegel RL, Ferlay J, Lortet-Tieulent J, Jemal A (2015). Global cancer statistics, 2012. CA Cancer J Clin.

[3] Zuo TT, Zheng RS, Zhang SW, Zeng HM, Chen WQ (2015). Incidence and mortality of liver cancer in China in 2011. Chinese journal of cancer.

[4] Panayiotidis MI (2014). Cancer epigenetics as biomarkers of clinical significance. Cancer letters.

[5] Udali S, Guarini P, Moruzzi S, Ruzzenente A, Tammen SA, Guglielmi A, Conci S, Pattini P, Olivieri O, Corrocher R, Choi SW, Friso S (2015). Global DNA methylation and hydroxymethylation differ in hepatocellular carcinoma and cholangiocarcinoma and relate to survival rate. Hepatology.

[6] Rich N, Singal AG (2014). Hepatocellular carcinoma tumour markers: current role and expectations. Best Pract Res Clin Gastroenterol.

[7] Fonseca AL, Cha CH (2014). Hepatocellular carcinoma: a comprehensive overview of surgical therapy. J Surg Oncol.

[8] Richter AM, Pfeifer GP, Dammann RH (2009). The RASSF proteins in cancer; from epigenetic silencing to functional characterization. Biochimica et biophysica acta.

[9] Sherwood V, Recino A, Jeffries A, Ward A, Chalmers AD (2010). The N-terminal RASSF family: a new group of Ras-association-domain-containing proteins, with emerging links to cancer formation. Biochem J.

[10] Underhill-Day N, Hill V, Latif F (2011). N-terminal RASSF family: RASSF7-RASSF10. Epigenetics.

[11] Chan JJ, Katan M (2013). PLCvarepsilon and the RASSF family in tumour suppression and other functions. Adv Biol Regul.

[12] Richter AM, Walesch SK, Wurl P, Taubert H, Dammann RH (2012). The tumor suppressor RASSF10 is upregulated upon contact inhibition and frequently epigenetically silenced in cancer. Oncogenesis.

[13] Richter AM, Haag T, Walesch S, Herrmann-Trost P, Marsch WC, Kutzner H, Helmbold P, Dammann RH (2013). Aberrant Promoter Hypermethylation of RASSF Family Members in Merkel Cell Carcinoma. Cancers (Basel).

[14] Shinawi T, Hill V, Dagklis A, Baliakas P, Stamatopoulos K, Agathanggelou A, Stankovic T, Maher ER, Ghia P, Latif F (2012). KIBRA gene methylation is associated with unfavorable biological prognostic parameters in chronic lymphocytic leukemia. Epigenetics.

[15] Hesson LB, Dunwell TL, Cooper WN, Catchpoole D, Brini AT, Chiaramonte R, Griffiths M, Chalmers AD, Maher ER, Latif F (2009). The novel RASSF6 and RASSF10 candidate tumour suppressor genes are frequently epigenetically inactivated in childhood leukaemias. Mol Cancer.

[16] Wei Z, Chen X, Chen J, Wang W, Xu X, Cai Q (2013). RASSF10 is epigenetically silenced and functions as a tumor suppressor in gastric cancer. Biochemical and biophysical research communications.

[17] Wang Y, Ma T, Bi J, Song B, Zhou Y, Zhang C, Gao M (2014). RASSF10 is epigenetically inactivated and induces apoptosis in lung cancer cell lines. Biomed Pharmacother.

[18] Lu D, Ma J, Zhan Q, Li Y, Qin J, Guo M (2014). Epigenetic silencing of RASSF10 promotes tumor growth in esophageal squamous cell carcinoma. Discov Med.

[19] Schagdarsurengin U, Richter AM, Wohler C, Dammann RH (2009). Frequent epigenetic inactivation of RASSF10 in thyroid cancer. Epigenetics.

[20] Helmbold P, Richter AM, Walesch S, Skorokhod A, Marsch W, Enk A, Dammann RH (2012). RASSF10 promoter hypermethylation is frequent in malignant melanoma of the skin but uncommon in nevus cell nevi. J Invest Dermatol.

[21] Dansranjavin T, Wagenlehner F, Gattenloehner S, Steger K, Weidner W, Dammann R, Schagdarsurengin U (2012). Epigenetic down regulation of RASSF10 and its possible clinical implication in prostate carcinoma. Prostate.

[22] Li Z, Chang X, Dai D, Deng P, Sun Q (2014). RASSF10 is an epigenetically silenced tumor suppressor in gastric cancer. Oncology reports.

[23] Guo J, Yang Y, Yang Y, Linghu E, Zhan Q, Brock MV, Herman JG, Zhang B, Guo M (2015). RASSF10 suppresses colorectal cancer growth by activating P53 signaling and sensitizes colorectal cancer cell to docetaxel. Oncotarget.

[24] Hill VK, Underhill-Day N, Krex D, Robel K, Sangan CB, Summersgill HR, Morris M, Gentle D, Chalmers AD, Maher ER, Latif F (2011). Epigenetic inactivation of the RASSF10 candidate tumor suppressor gene is a frequent and an early event in gliomagenesis. Oncogene.

[25] Song K, Wu J, Jiang C (2013). Dysregulation of signaling pathways and putative biomarkers in liver cancer stem cells (Review). Oncology reports.

[26] Huang X, Ma J, Xu J, Su Q, Zhao J (2015). Simvastatin induces growth inhibition and apoptosis in HepG2 and Huh7 hepatocellular carcinoma cells *via* upregulation of Notch1 expression. Molecular medicine reports.

[27] Stoyanov E, Ludwig G, Mizrahi L, Olam D, Schnitzer-Perlman T, Tasika E, Sass G, Tiegs G, Jiang Y, Nie T, Kohler J, Schinazi RF, Vertino PM (2015). Chronic liver inflammation modifies DNA methylation at the precancerous stage of murine hepatocarcinogenesis. Oncotarget.

[28] Mizuno S, Chijiwa T, Okamura T, Akashi K, Fukumaki Y, Niho Y, Sasaki H (2001). Expression of DNA methyltransferases DNMT1, 3A, and 3B in normal hematopoiesis and in acute and chronic myelogenous leukemia. Blood.

[29] Kanai Y, Ushijima S, Kondo Y, Nakanishi Y, Hirohashi S (2001). DNA methyltransferase expression and DNA methylation of CPG islands and peri-centromeric satellite regions in human colorectal and stomach cancers. International journal of cancer.

[30] Park HJ, Yu E, Shim YH (2006). DNA methyltransferase expression and DNA hypermethylation in human hepatocellular carcinoma. Cancer letters.

[31] Oh BK, Kim H, Park HJ, Shim YH, Choi J, Park C, Park YN (2007). DNA methyltransferase expression and DNA methylation in human hepatocellular carcinoma and their clinicopathological correlation. Int J Mol Med.

[32] Fan H, Zhao ZJ, Cheng J, Su XW, Wu QX, Shan YF (2009). Overexpression of DNA methyltransferase 1 and its biological significance in primary hepatocellular carcinoma. World journal of gastroenterology.

[33] Nagai M, Nakamura A, Makino R, Mitamura K (2003). Expression of DNA (5-cytosin)-methyltransferases (DNMTs) in hepatocellular carcinomas. Hepatol Res.

[34] Ueda R, Suzuki T, Mino K, Tsumoto H, Nakagawa H, Hasegawa M, Sasaki R, Mizukami T, Miyata N (2009). Identification of cell-active lysine specific demethylase 1-selective inhibitors. J Am Chem Soc.

[35] Datta J, Ghoshal K, Sharma SM, Tajima S, Jacob ST (2003). Biochemical fractionation reveals association of DNA methyltransferase (Dnmt) 3b with Dnmt1 and that of Dnmt 3a with a histone H3 methyltransferase and Hdac1. J Cell Biochem.

[36] Weisenberger DJ, Velicescu M, Cheng JC, Gonzales FA, Liang G, Jones PA (2004). Role of the DNA methyltransferase variant DNMT3b3 in DNA methylation. Mol Cancer Res.

[37] Qiu X, Zhang L, Lu S, Song Y, Lao Y, Hu J, Fan H (2014). Upregulation of DNMT1 mediated by HBx suppresses RASSF1A expression independent of DNA methylation. Oncology reports.

[38] Shen L, Ahuja N, Shen Y, Habib NA, Toyota M, Rashid A, Issa JP (2002). DNA methylation and environmental exposures in human hepatocellular carcinoma. J Natl Cancer Inst.

[39] Hlady RA, Tiedemann RL, Puszyk W, Zendejas I, Roberts LR, Choi JH, Liu C, Robertson KD (2014). Epigenetic signatures of alcohol abuse and hepatitis infection during human hepatocarcinogenesis. Oncotarget.

[40] Yang P, Ma J, Zhang B, Duan H, He Z, Zeng J, Zeng X, Li D, Wang Q, Xiao Y, Liu C, Xiao Q, Chen L (2012). CpG site-specific hypermethylation of p16INK4alpha in peripheral blood lymphocytes of PAH-exposed workers. Cancer epidemiology, biomarkers & prevention.

[41] Feng Y, Xue WJ, Li P, Sha ZY, Huang H, Rui L, Li HX, Mao QS (2012). RASSF1A hypermethylation is associated with aflatoxin B1 and polycyclic aromatic hydrocarbon exposure in hepatocellular carcinoma. Hepato-gastroenterology.

[42] Wu HC, Wang Q, Yang HI, Tsai WY, Chen CJ, Santella RM (2013). Global DNA methylation in a population with aflatoxin B1 exposure. Epigenetics.

[43] Zhu YZ, Zhu R, Shi LG, Mao Y, Zheng GJ, Chen Q, Zhu HG (2010). Hepatitis B virus X protein promotes hypermethylation of p16(INK4A) promoter through upregulation of DNA methyltransferases in hepatocarcinogenesis. Exp Mol Pathol.

[44] Guo J, Fu Z, Wei J, Lu W, Feng J, Zhang S (2015). PRRX1 promotes epithelial-mesenchymal transition through the Wnt/beta-catenin pathway in gastric cancer. Medical oncology.

[45] Xu YF, Yi Y, Qiu SJ, Gao Q, Li YW, Dai CX, Cai MY, Ju MJ, Zhou J, Zhang BH, Fan J (2010). PEBP1 downregulation is associated to poor prognosis in HCC related to hepatitis B infection. Journal of hepatology.

[46] Mei H, Lian S, Zhang S, Wang W, Mao Q, Wang H (2014). High expression of ROR2 in cancer cell correlates with unfavorable prognosis in colorectal cancer. Biochemical and biophysical research communications.

[47] Xue WJ, Li C, Zhou XJ, Guan HG, Qin L, Li P, Wang ZW, Qian HX (2008). RASSF1A expression inhibits the growth of hepatocellular carcinoma from Qidong County. J Gastroenterol Hepatol.

