# Supplementary material for: RASSF10 is an epigenetically inactivated tumor suppressor and independent prognostic factor in hepatocellular carcinoma
Source: Oncotarget. 2015 Dec 18;7(4):4279–97. doi: 10.18632/oncotarget.6654 (PMC4826205; doi:10.18632/oncotarget.6654)
Supplement: Supplementary file 1 [file oncotarget-07-4279-s001.pdf]

RASSF10 is an epigenetically inactivated tumor suppressor and independent prognostic factor in hepatocellular carcinoma

Supplementary Material

Immunofluorescence

Cells were grown on glass cover slips to 40%-50% confluence, and then fixed, permeabilized, and blocked. Cells were then incubated with primary monoclonal antibodies against E-cadherin and β-catenin (Cell Signaling Technology, Boston, MA) overnight at 4°C. The next day, slides were washed and incubated with TRITC-conjugated anti-rabbit IgG (sc-3841) (Santa Cruz Biotechnology, Dallas, TX). Cells were counterstained with 4'-6-diamidino-2-phenylindole (DAPI) to visualize cell nuclei, and were visualized by Leica Confocal Laser Scanning Microscope (Leica， Germany).

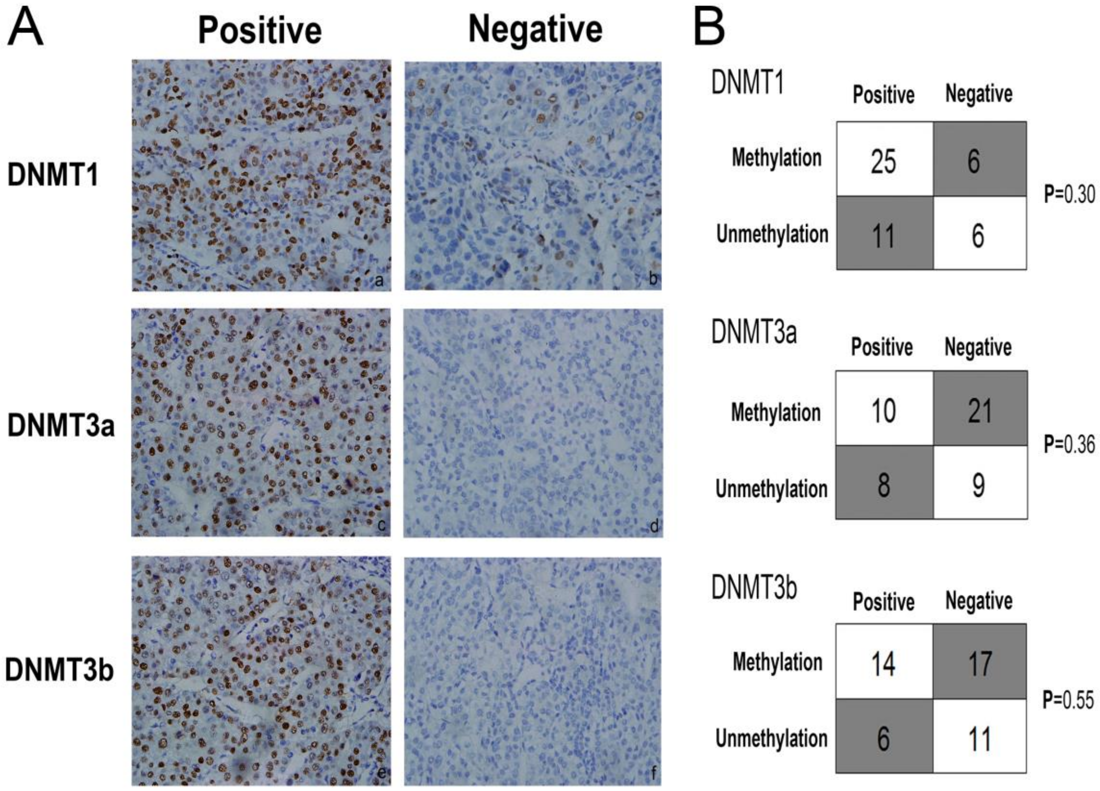

Supplementary Figure S1: DNMT protein expression and relationship with methylation in HCC. Immunohistochemical stain results **A.** for DNMT1 (a and b), DNMT3a (c and d) and DNMT3b (e and f) proteins in HCC. Positive expression and negative expression are seen in these six cases. Bar 100μm ×400. The correlation analysis **B.** of DNMT (DNMT1, DNMT3a and DNMT3b) protein expression and methylation in 48 HCC samples.

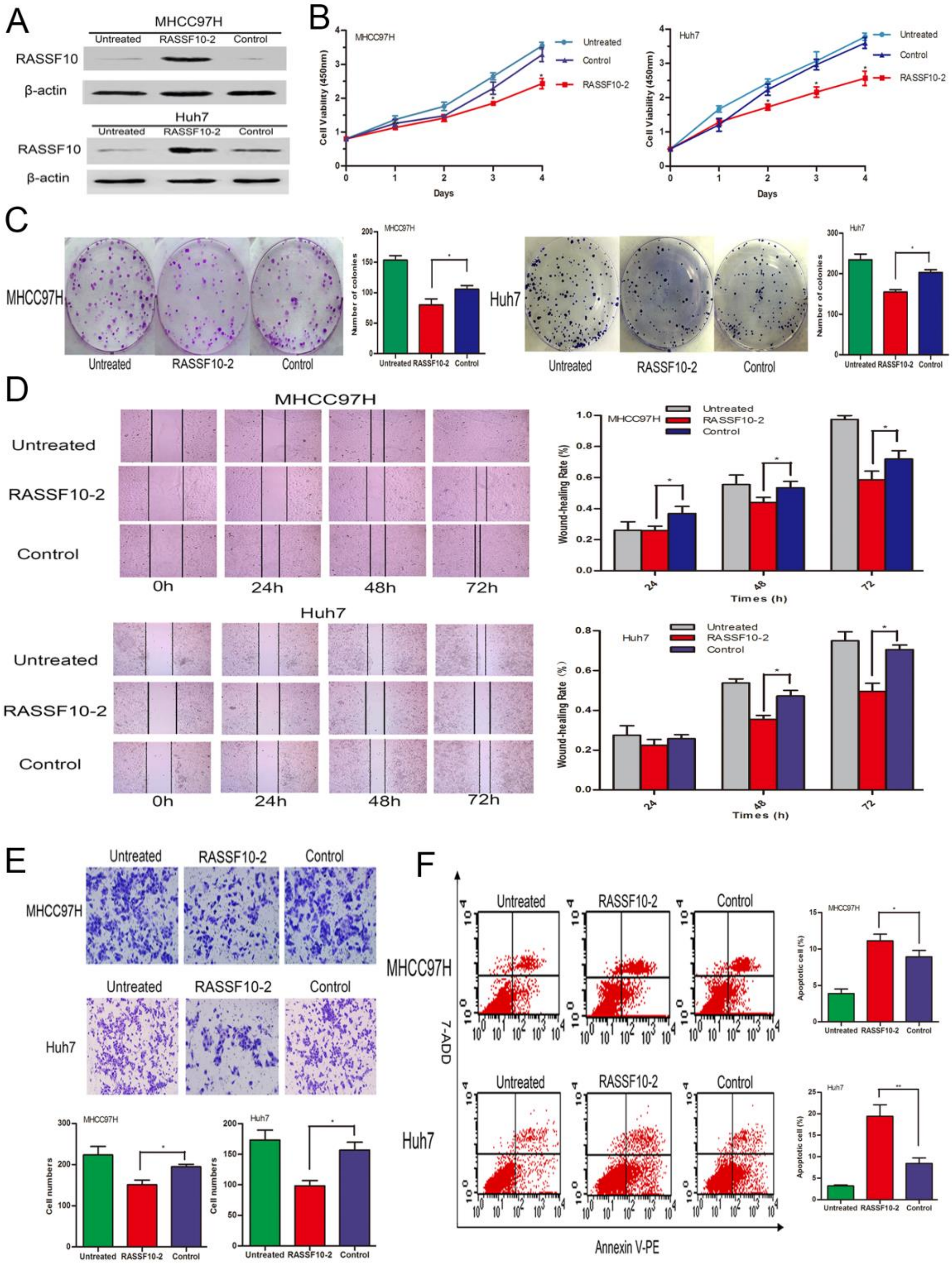

Supplementary Figure S2: Influence of RASSF10 on HCC cell growth, migration, invasion and apoptosis. RASSF10 protein levels **A.** in lentiviral transfected MHCC97H and Huh7 cells. One subline stably expressed RASSF10, and the control expressed empty lentiviral plasmid. MHCC97H and Huh7 cells with no

lentiviral transfection served as untreated controls. CCK-8 **B.** and colony formation assays **C.** were employed to detect the effect of RASSF10 on the growth of the recombinant MHCC97H and Huh7 cells. Wound healing assays **D.** and invasion behavior **E.** of the recombinant MHCC97H and Huh7 cells. Percentage of apoptotic cells **F.** in the recombinant MHCC97H and Huh7 cells. Quantitative analyses of apoptotic cell numbers are plotted as the mean ± SD of three independent experiments. *, $P<0.05$.

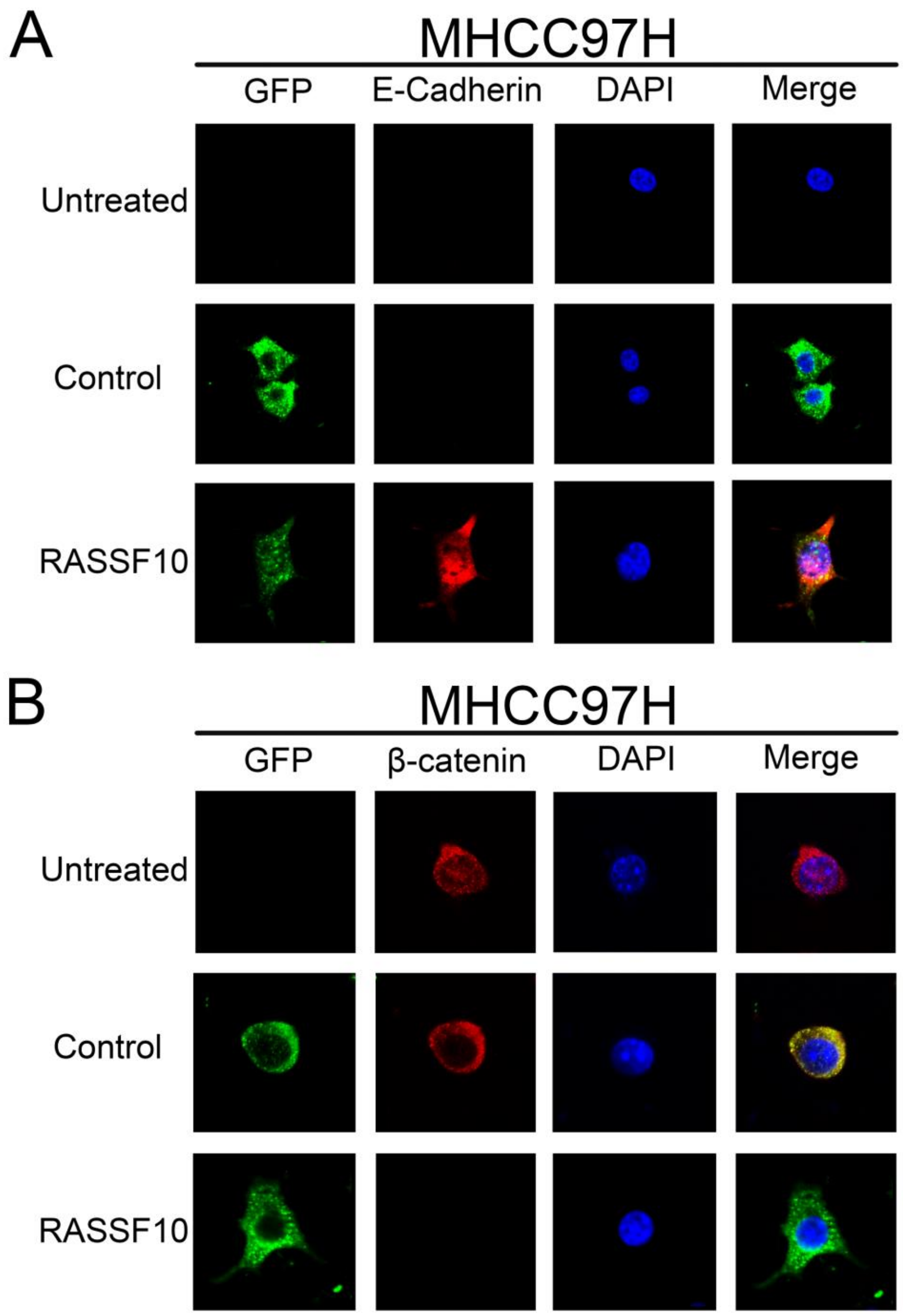

Supplementary Figure S3: Immunofluorescent staining of E-cadherin and β-catenin in lentivirus-transfected MHCC97H cells. E-cadherin **A.** was upregulated, and β-catenin **B.** was downregulated in recombinant MHCC97H cells. One subline stably expressed RASSF10, and the control expressed empty lentiviral plasmid. MHCC97H cells with no lentiviral transfection served as untreated controls.
